# Researches on Phthisis, Anatomical, Pathological, and Therapeutical

**Published:** 1844-10

**Authors:** 


Art. II.—1. *Researches on Phthisis, Anatomical, Pathological, and Therapeutical.* By P. C. A. Louis, m.d. &c. Second Edition, considerably enlarged. Translated by W. H. Walshe, m.d., &c.—*London; Printed for the Sydenham Society*, 1844. 8vo, pp. 572.

2. *Thomæ Sydenham*, m.d. *Opera Omnia:* Edidit Gulielmus Alex. Greenhill, m.d.—*Londini, impensis Societatis Sydenhamianæ*, 1844. 8vo, pp. 668.

We stated on a former occasion (Br. and For. Med. Rev. vol. XVII, p. 522), that it was not our intention to *review*, in the ordinary sense of the word, any of the publications of the Sydenham Society, and for the two obvious and sufficient reasons—that the works are not published for sale, and will come into the hands and libraries of almost every one of our readers. Lest, however, it should be supposed that total silence on our part might imply indifference to the interests of the Society, or disapproval of the works published by it, we think it necessary to say a few words respecting the two volumes whose titles we have just transcribed.

I. We regard the fact of two thousand members of the profession becoming, as members of the Sydenham Society, *possessors* of the classical work of Louis, in a language intelligible to all, as one of great importance. This work, to use the words of Dr. Walshe, is "universally admitted to contain the most profound exposition of the natural history of chronic disease, of which the literature of any age or country can boast;" and as it cannot be doubted that much of the bad practice, and of the false appreciation of the value of remedies in chronic ailments generally, and especially in phthisis, originates in ignorance of the real natural course of diseases, we look forward to corresponding benefit from the lesson that will be thus extensively taught. Had Dr. Ramadge,

Dr. Cronin, Dr. Turnbull, Dr. Hastings, and the host of other blockheads who of late years have come forward as curers of consumption, been acquainted with the natural history of this malady, they would never (supposing them honest) have themselves credited the results they have attempted to pass on the profession and the public as facts. And, although we do not reckon on such men being teachable, we believe that many honorable practitioners will rise from the study of Louis with much juster views of the natural progress of diseases, and of the true value of therapeutic agents, than they possessed before. It is this prevalent ignorance of the *natural history of diseases*, that gives to homœopathy its strong hold in public estimation, and enables it to defy the arguments of its professional opponents. If we knew what *nature* could do, or could not do, in any particular disease, then we could pronounce of the true value of the ten-trillionth part of a grain of any common vegetable juice in curing it; but while we are ignorant of this, we cannot say, with certainty, that the conceivable cure was not effected by the inconceivable dose; though, at the same time, we can say, with the utmost truth, that—thanks to the same ignorance of nature's powers —the homœopathist has not one ten-trillionth of a tittle of proof that such was actually the case. Before we can believe his doctrines, he must show us that nature cannot do what he professes to do.

Our readers are probably aware that Dr. Cowan of Reading published an excellent translation of the first edition of this work. His engagements prevented him acceding to the request of the council of the Sydenham Society, that he should undertake the translation of the new edition. The council were fortunate in finding such a substitute as Dr. Walshe. He has performed the task of translator in an admirable manner; and to all future time the *Researches on Phthisis* will remain, in every sense of the word, one of the English medical classics.

II. Of the new edition of the Works of Sydenham we need say but little. As no one can doubt of the inestimable value of the book, so no one can doubt of the propriety of its being one of the new society's earliest publications. The present volume contains the Latin version only; the English edition will be published next year. As might be expected, from the well-known erudition and talents of Dr. Greenhill, we have here an edition of our great countryman's writings, of the utmost accuracy and elegance. It is, without question, superior to any that has preceded it. The text has been revised throughout with the most scrupulous care, and illustrated by the most judicious notes and references. These are the more to be commended, that they occupy a very small space. A most valuable addition is that of the corresponding modern names of the old medicines and formularies; in the preparation of which the editor acknowledges the valuable assistance of Drs. Pereira and Royle.

The two volumes now noticed, and Dr. Babington's Hecker, noticed in a former Number, constitute the first yar's publications of the Sydenham Society. In looking at their intrinsic value as literary productions, at their beauty and elegance as specimens of typography, and at their relative commercial value as mere material books, we have proof that all our anticipations of the great importance of the Sydenham Society to the medical profession have been already more than realized ; and we entertain no manner of doubt that, when it is generally known that three such volumes as these now on our table (the selling price of which in the shops would be *at least* two guineas) are promised to the members, in return for their annual subscriptions, the supporters of the Society will soon be doubled or tripled. The present number of members is about two thousand.

